# Genome-wide identification and functional analysis of circRNAs in *Trichophyton rubrum* conidial and mycelial stages

**DOI:** 10.1186/s12864-021-08184-y

**Published:** 2022-01-04

**Authors:** Xingwei Cao, Xingye Xu, Jie Dong, Ying Xue, Lilian Sun, Yafang Zhu, Tao Liu, Qi Jin

**Affiliations:** NHC Key Laboratory of Systems Biology of Pathogens, Institute of Pathogen Biology, Chinese Academy of Medical Sciences & Peking Union Medical College, Beijing, P. R. China

**Keywords:** *Trichophyton rubrum* (*T. rubrum*), High-throughput sequencing, Circular RNA (circRNA), microRNA (miRNA), Target gene

## Abstract

**Background:**

Circular RNAs (circRNAs) are a group of noncoding RNAs that participate in gene expression regulation in various pathways. The essential roles of circRNAs have been revealed in many species. However, knowledge of circRNAs in fungi is still not comprehensive.

**Results:**

*Trichophyton rubrum* (*T. rubrum*) is considered a model organism of human pathogenic filamentous fungi and dermatophytes. In this study, we performed a genome-wide investigation of circRNAs in *T. rubrum* based on high-throughput sequencing and ultimately identified 4254 circRNAs. Most of these circRNAs were specific to the conidial or mycelial stage, revealing a developmental stage-specific expression pattern. In addition, 940 circRNAs were significantly differentially expressed between the conidial and mycelial stages. PCR experiments conducted on seven randomly selected differentially expressed (DE-) circRNAs confirmed the circularized structures and relative expression levels of these circRNAs. Based on their genome locations, most circRNAs originated from intergenic regions, unlike those in plants and animals. Furthermore, we constructed circRNA-miRNA-mRNA regulatory networks that included 661 DE-circRNAs targeting 140 miRNAs and further regulating 2753 mRNAs. The relative expression levels of two randomly selected circRNA-miRNA-mRNA axes were investigated by qRT-PCR, and the competing endogenous RNA (ceRNA) network theory was validated. Functional enrichment analysis of the target genes suggested that they were significantly involved in posttranscriptional processes and protein synthesis as well as some small-molecule metabolism processes. CircRNAs are relatively more conserved in closely related dermatophytes but rarely conserved in distantly related species. Tru_circ07138_001 is a highly conserved circRNA that was conserved in all ten dermatophytes analyzed in our study and three distantly related species. Its host gene TERG_07138 was also highly conserved in two of these distantly related species *Gallus gallus* and *Caenorhabditis elegans*. The specific role of this circRNA deserves further exploration.

**Conclusions:**

Our study is the first to provide a global profile of circRNAs in *T. rubrum* as well as dermatophytes. These results could serve as valuable resources for research on circRNA regulatory mechanisms in fungi and reveal new insights for further investigation of the physical characteristics of these significant human fungal pathogens.

**Supplementary Information:**

The online version contains supplementary material available at 10.1186/s12864-021-08184-y.

## Background

Circular RNAs (circRNAs) are a group of endogenous noncoding RNAs (ncRNAs) that are each composed of a single-stranded covalently bonded ring structure [1, 2]. In 1976, Sanger et al. first discovered the existence of circRNAs in plant viroids [3]. In the following decades, circRNAs were also detected in other viruses and human cells [4, 5]. Due to the limitations of sequencing and analysis methods, circRNAs were originally mistaken as rare viroid-characteristic or useless RNAs produced by abnormal RNA splicing [6]. Recently, studies on circRNAs have achieved great progress given the rapid advances in high-throughput sequencing technology; the development of a growing number of circRNA identification tools, such as CircInteractome [7], circRNA_finder [8], CircRNA Identifier (CIRI) [9], find_circ [10] and CIRCexplorer [11]; and the establishment of circRNA annotation databases, including CircNet [12], Circ2Traits [13] and deepBase v2.0 [14].

CircRNAs are generated by back-splicing of precursor mRNA (pre-mRNA) [1]. In contrast to canonical splicing, which joins the upstream 5′ splice (donor) site with a downstream 3′ splice (acceptor) site to form a linear RNA transcript, back-splicing connects a downstream 5′ splice site with an upstream 3′ splice site, forming a covalently closed circRNA transcript [15]. Due to their ring structures, circRNAs are more stable and less prone to degradation than linear transcripts [16]. CircRNAs are essential transcription regulators that are considered microRNA (miRNA) sponges that competitively regulate target gene expression [17]. In addition, circRNA biogenesis may compete with host pre-mRNA linear splicing, and some circRNAs with internal ribosomal entry sites (IRESs) and N^6^-methyladenosine (m^6^A) have translational potential [1, 18]. The essential roles of circRNAs have been illustrated in many species, including humans [19], mice [20], zebrafish [21], *Caenorhabditis elegans* [22], Drosophila [8], *Saccharomyces cerevisiae* [23], *Arabidopsis thaliana* [24], rice [25] and soybean [26].

CircRNAs in fungi have been researched in only a few studies. In 2018, Guo et al. identified 551 circRNAs in *Ascosphaera apis*, most of which ranged in length from 200 to 600 nt, unlike circRNAs in animals and plants. These circRNAs play vital roles in metabolism, environmental stress and gene expression [27]. In 2018, Yuan et al. detected 2721 and 5840 circRNAs in conidial and mycelial samples, respectively, of the plant pathogenic fungus *Magnaporthe oryzae*, which infects rice. The host genes of circRNAs in conidia were involved mainly in the synthesis of storage products for rice infection, while those of circRNAs in mycelia were involved primarily in basic metabolism for the promotion of normal fungal growth [28]. In addition, Guo et al. identified 204 circRNAs in spore samples of the microsporidium species *Nosema ceranae*, and circRNA-miRNA-mRNA regulatory networks showed that the target genes were engaged in various metabolic pathways, indicating that these circRNAs might play regulatory roles in *N. ceranae* spores [29]. However, knowledge about circRNAs in fungi remains from sufficient.

*Trichophyton rubrum* (*T. rubrum*) is the most common pathogen responsible for fungal infections worldwide and has been studied as a model organism of dermatophytes and human pathogen filamentous fungi [30–32]. In addition to dermatomycosis, deep infections caused by *T. rubrum* have been occasionally reported and threaten the lives of immune-deficient patients [33, 34]. The incidence of infections caused by *T. rubrum* continues to rise, and these infections are receiving increasing attention [35]. Resistance to antifungal reagents and frequent relapses are also great concerns in therapy [36]. *T. rubrum* has two major developmental stages in its life cycle: the conidial stage, in which the fungus adheres to the stratum corneum of skin, and the mycelial stage, in which the fungus penetrates and destroys the superficial layers [37]. The biological characteristics and pathogenic mechanisms of *T. rubrum* have been extensively studied at the molecular level, which has improved our understanding of this fungus [38–43]. The characteristics and roles of circRNAs in *T. rubrum* remain unknown.

In the current study, we performed global profiling of circRNAs in *T. rubrum* based on high-throughput RNA sequencing (RNA-seq). A total of 4254 circRNAs were identified in both conidial and mycelial stages, and 940 circRNAs were considered to be significantly differentially expressed between the two stages. With further bioinformatics analysis, the genomic location, alternative splicing (AS) pattern, conservation and regulatory roles of circRNAs were also investigated. These results provide new clues for research on circRNA regulation mechanisms in fungi and could serve as a foundation for further studies on these medically important pathogenic fungi.

## Results

### Overview of high-throughput sequencing data for *T. rubrum*

To identify distinct patterns of circRNAs in the two major growth stages of *T. rubrum*, we constructed cDNA libraries of conidia and mycelia with three biological replicates and sequenced them with an Illumina HiSeq platform. After the adaptors were trimmed and the low-quality reads or reads with unknown (N) bases were filtered out, averages of 125.1749 million and 124.6443 million clean reads were obtained for the conidial and mycelial stages, respectively (Table 1). All clean reads were mapped to the *T. rubrum* reference genome, and the mean mapping rate for the six samples was 97.6%. In addition, the average values of Pearson’s correlation coefficient were 0.9385 and 0.8239 for circRNA expression levels in conidia and mycelia, respectively (Additional file 1: Fig. S1), highlighting good consistency among the three replicates in each stage. Together, these results suggest the reliability and repeatability of our sequenced data.

**Table 1:** Summary of RNA-seq data for *T. rubrum* conidial and mycelial samples

Sample	Clean reads	GC (%)	Q30 (%)	Mapped reads	Mapping rate (%)
Conidia_1	122.5520 M	46.58	90.92	121.0295 M	98.76%
Conidia_2	126.8353 M	46.93	90.46	123.7974 M	97.61%
Conidia_3	126.1375 M	46.21	91.31	120.9987 M	95.93%
Mycelia_1	124.3186 M	45.98	91.34	121.7001 M	97.90%
Mycelia_2	127.2742 M	44.71	89.99	123.7542 M	97.24%
Mycelia_3	122.3400 M	45.51	90.68	120.1093 M	98.18%

### Identification and characterization of circRNAs

A total of 4254 circRNAs (Additional file 2: Table S1) were identified in *T. rubrum*, including 3980 in conidia and 778 in mycelia. Most of the identified circRNAs were growth stage specific; 3476 circRNAs were specific to conidia, and 274 circRNAs were specific to mycelia.

To obtain an overview of circRNA distribution within the *T. rubrum* genome, we investigated the locations of circRNAs on each genomic contig (Table 2). The numbers of circRNAs on distinct genomic contigs were significantly different. This difference may have been due to the length differences of distinct contigs.

**Table 2:** Distribution of circRNAs and back-spliced reads in the whole genome of *T. rubrum*

Genomic region	CircRNA number	CircRNA percentage	No. of back-spliced reads	Read percentage
NW_003456399.1	1	0.02%	4	0.01%
NW_003456411.1	7	0.16%	19	0.07%
NW_003456412.1	29	0.68%	101	0.38%
NW_003456413.1	10	0.24%	128	0.48%
NW_003456414.1	32	0.75%	149	0.55%
NW_003456415.1	36	0.85%	187	0.69%
NW_003456416.1	37	0.87%	144	0.53%
NW_003456417.1	93	2.19%	566	2.10%
NW_003456418.1	192	4.51%	1008	3.73%
NW_003456419.1	152	3.57%	854	3.16%
NW_003456420.1	232	5.45%	1450	5.37%
NW_003456421.1	243	5.71%	1155	4.28%
NW_003456422.1	332	7.80%	2167	8.03%
NW_003456423.1	288	6.77%	1585	5.87%
NW_003456424.1	353	8.30%	2233	8.27%
NW_003456425.1	432	10.16%	3943	14.60%
NW_003456426.1	457	10.74%	2523	9.35%
NW_003456427.1	457	10.74%	2347	8.69%
NW_003456428.1	853	20.06%	6341	23.49%
NW_003456429.1	18	0.43%	95	0.35%
Total	4254	100.00%	26,999	100.00%

**Fig. 1:**
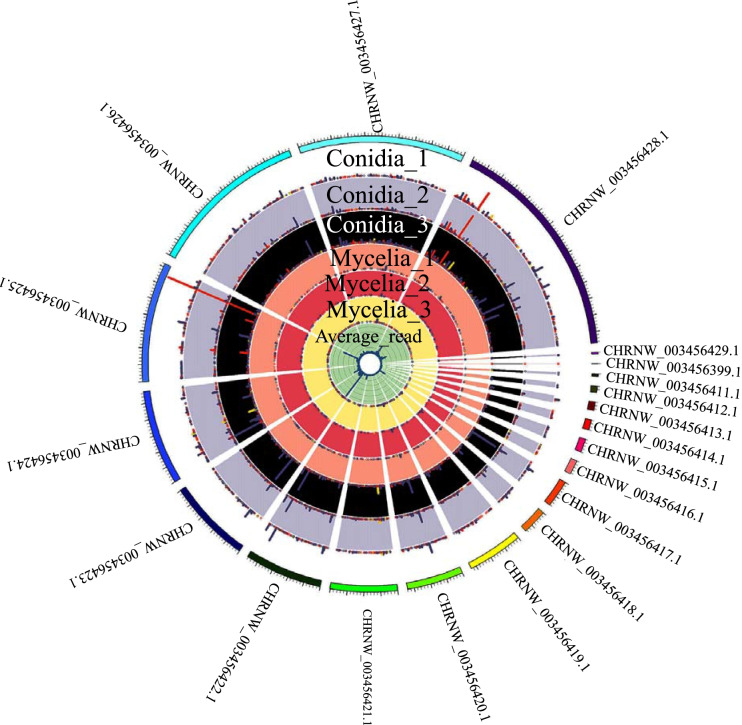
The Circos plot shows the distribution and abundance of circRNAs in the whole genome. The red bar represents exonic circRNAs, the yellow bar represents intronic circRNAs and the blue bar represents intergenic circRNAs. The height of each bar indicates the read number for each circRNA. The outermost layer represents genomic contigs. The six layers in the middle represent the three replicates in each stage, including conidia replicate 1, conidia replicate 2, conidia replicate 3, mycelia replicate 1, mycelia replicate 2 and mycelia replicate 3. The innermost layer shows the number of average junction reads per circRNA of these six samples

We also created a Circos plot to directly visualize the distribution of circRNAs in the whole genome of *T. rubrum.* As shown in Fig. 1, circRNAs were evenly distributed on all contigs, suggesting that circRNAs have no preference for genomic regions.

**Fig. 2:**
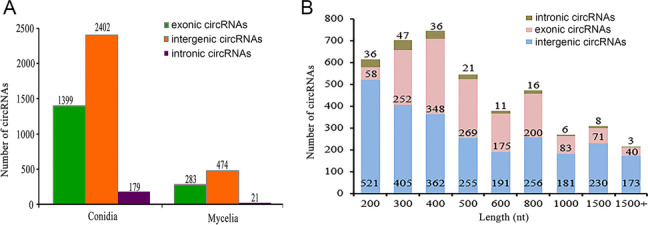
Type and length of all the identified circRNA. **a** The number of each type of circRNA in the conidial and mycelial stages. **b** Length distribution of intronic, intergenic and exonic circRNAs

In addition, the identified circRNAs could be classified into three groups, including exonic, intronic and intergenic circRNAs, according to their genomic locations. Intergenic circRNAs were the most abundant (60.5%), followed by exonic circRNAs (35.2%) and intronic circRNAs (4.3%). When the conidial and mycelial stages were compared, these three kinds of circRNAs showed similar proportions in the distribution (Fig. 2a).

The lengths of circRNAs varied greatly. The longest circRNA was 196,395 nt, and the shortest was 20 nt. As shown in Fig. 2b, 61.36% of circRNAs were shorter than 500 nt, 26.3% were within 500–1000 nt, and 12.34% were longer than 1000 nt. The average length of all the identified circRNAs was 639 nt.

### Identification of differentially expressed (DE-) circRNAs

**Fig. 3:**
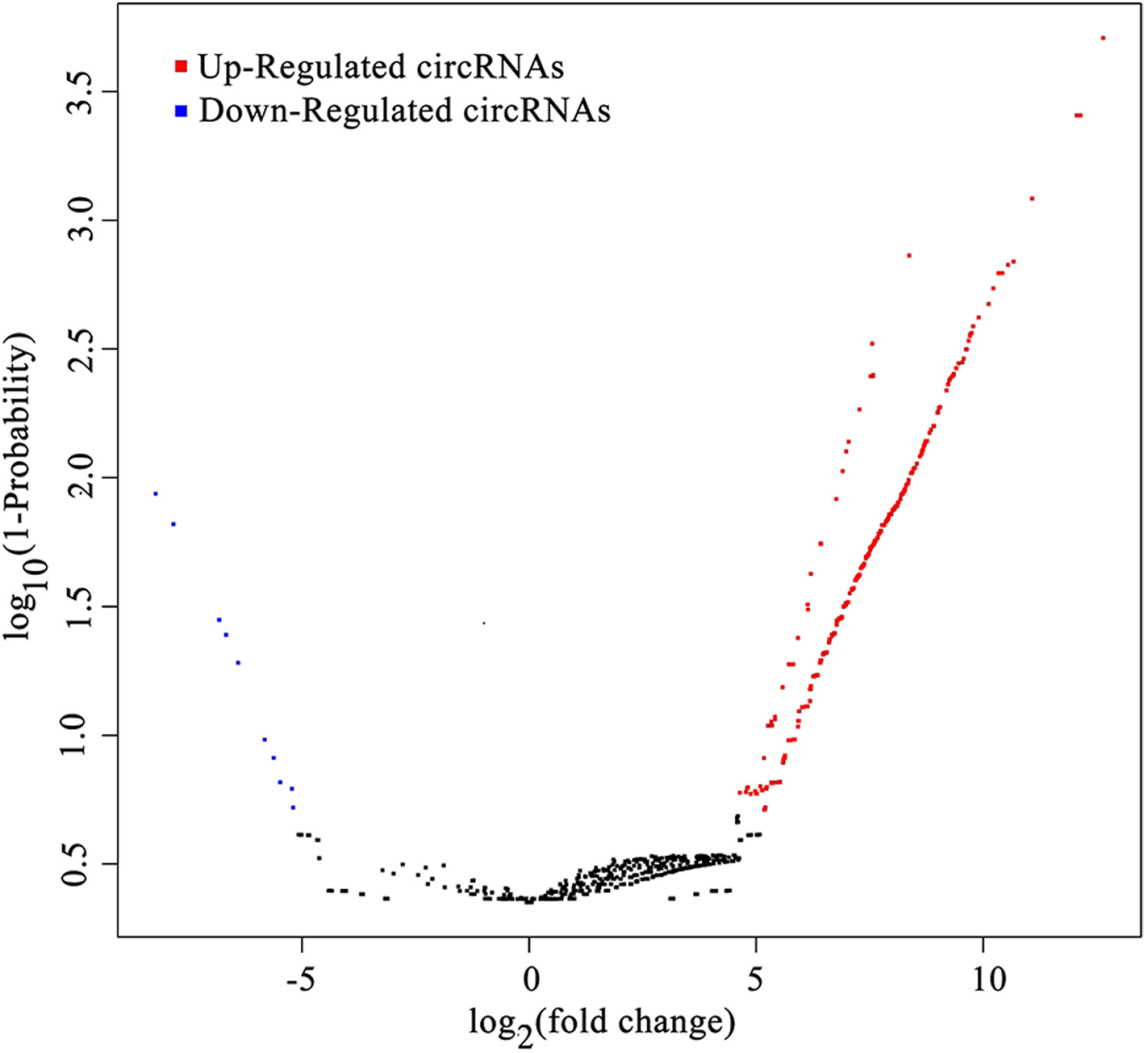
Volcano plot of circRNA relative expression levels in the conidial vs. mycelial stage. Red dots represent up-regulated circRNAs and blue dots represent down-regulated circRNAs in conidia vs. mycelia. Black dots represent circRNAs that are not significantly differentially expressed between the two stages

CircRNAs identified in the conidial and mycelial stages were compared to identify DE-circRNAs. Based on the criteria of |log_2_ (fold change)| ≥1 and probability ≥0.8 [44, 45], 940 circRNAs were considered to be significantly differentially expressed, including 930 up-regulated and 10 down-regulated circRNAs in the conidial vs. mycelial stage (Fig. 3). Detailed information on these DE-circRNAs is described in Additional file 3: Table S2. The most significantly up-regulated circRNAs were Tru_circ00222_002, Tru_circ_sc2.12_00022 and Tru_circ_sc2.5_00054, which had |log_2_ (fold change)| values of 12.64, 12.14 and 12.12, respectively, while the most significantly down-regulated circRNAs were Tru_circ05540_001, Tru_circ08130_001, and Tru_circ_sc2.6_00055, which had |log_2_ (fold change)| values of 8.23, 7.83 and 6.82, respectively.

Hierarchical cluster analysis additionally showed significantly different expression patterns between up-regulated and down-regulated circRNAs, and suggested good consistency among the three replicates (Additional file 1: Fig. S2).

### Validation of the circularized structures and relative expression levels of circRNAs

**Fig. 4:**
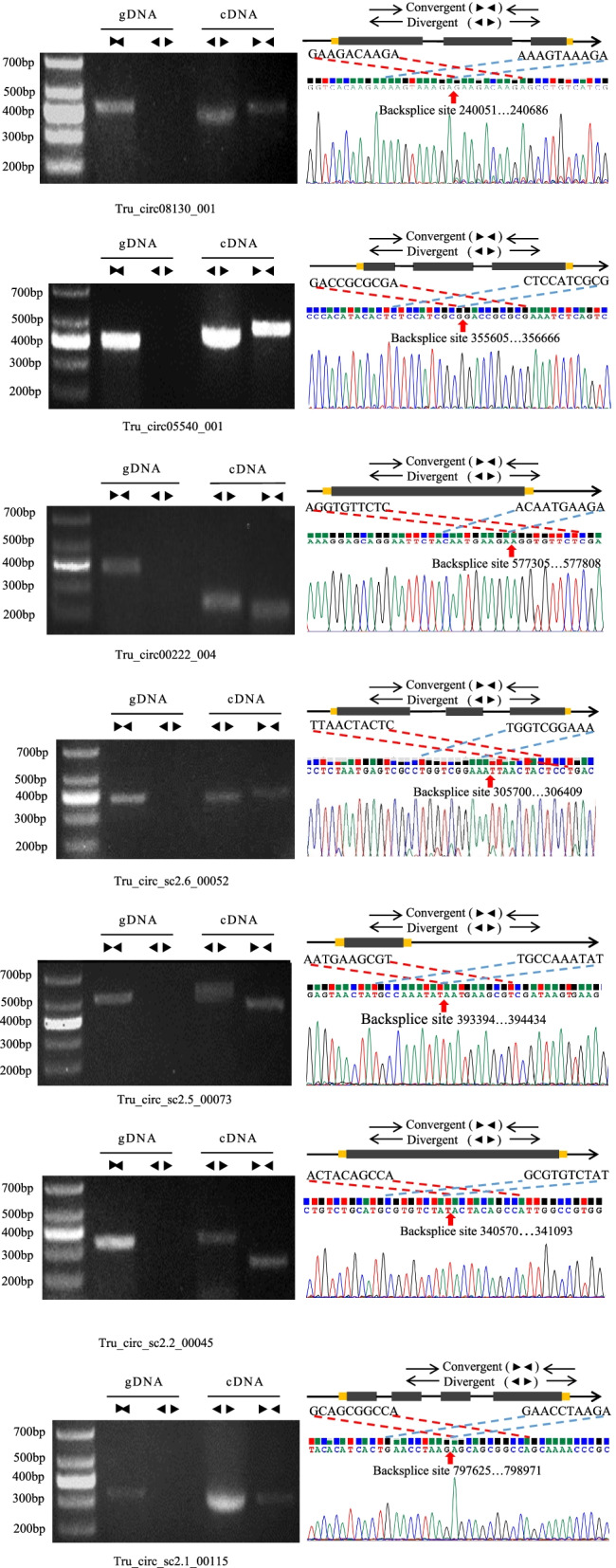
Validation of the circularized structure of circRNAs. Seven randomly selected DE-circRNAs were confirmed by PCR experiments with divergent and convergent primers. The amplification products were tested by agarose gel electrophoresis. Each cropped gel shows the bands that were amplified with divergent and convergent primers of both circRNA and gDAN control. The back-splicing junctions of circRNA were further confirmed by Sanger sequencing

To validate that the circRNAs identified in our study were circularized after splicing, we performed PCR experiments on seven randomly selected DE-circRNAs. Seven pairs of convergent and divergent primers were designed for these circRNAs. Theoretically, divergent primers designed to span circRNA back-splicing junctions should specifically amplify circularized RNAs, while convergent primers used as positive controls should amplify linear sequences of both the transcript and genome. As shown in Fig. 4 and Additional file 1: Fig. S3, all reactions using the cDNA template yielded amplification products of the expected sizes with both convergent and divergent primers. Meanwhile, amplification in the control reaction using the gDNA template could only be achieved with the convergent primers; the corresponding bands were not obtained with the divergent primers. To further confirm the fragments amplified by divergent primers spanning the expected back-splicing sites, these seven PCR-amplified products were subjected to Sanger sequencing. All of the sequencing results validated the expected splicing sites.

In addition, we verified the relative expression levels of these seven DE-circRNAs via qRT-PCR using divergent primers. Based on the results of high-throughput sequencing, five circRNAs, Tru_circ00222_004, Tru_circ_sc2.6_00052, Tru_circ_sc2.5_00073, Tru_circ_sc2.2_00045 and Tru_circ_sc2.1_00115, were up-regulated in conidia vs. mycelia, while two circRNAs, Tru_circ08130_001 and Tru_circ05540_001, were down-regulated. As shown in Additional file 1: Fig. S4, the qRT-PCR results for all of these circRNAs exhibited expression patterns consistent with those indicated by the high-throughput sequencing data. All of these results demonstrate that the circRNAs identified in our study are highly reliable.

### Identification of circRNA host genes and AS events

Among all the identified circRNAs, 1680 circRNAs were mapped to 1055 host genes. Among the mapped circRNAs, 326 DE-circRNAs corresponded to 249 host genes. To present a comprehensive landscape of circRNA origination, we performed GO and KEGG enrichment analyses for the host genes of all circRNAs (Additional file 4: Table S3, Additional file 5: Table S4, Additional file 1: Fig. S5, S6) and of DE-circRNAs (Additional file 6: Table S5, Additional file 7: Table S6 and Additional file 1: Fig. S7, S8). The results showed that the host genes were involved in various metabolic and biosynthetic processes.

In most instances, one host gene corresponded to one circRNA. In some cases, a host gene corresponded to two or more circRNAs. For instance, in our study, the TERG_08844 gene (Gene ID: 10372936) corresponded to 15 circRNAs. We also found that DE-circRNAs corresponding to identical host genes all showed consistent relative expression differences in the conidial vs. mycelial stage (i.e., they were all up-regulated or down-regulated).

**Fig. 5:**
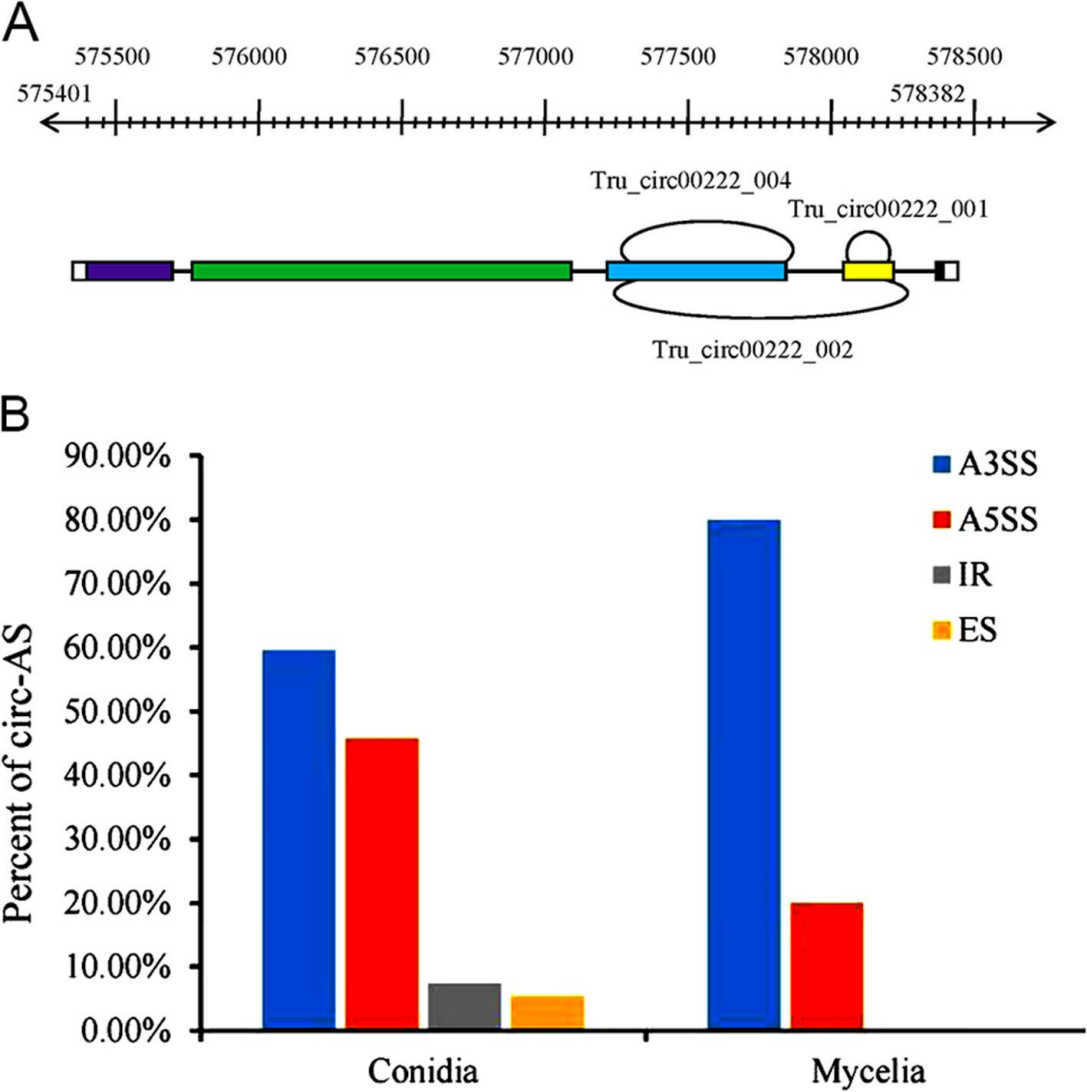
Alternative splicing events in *T. rubrum*. **a** Schematic chart illustrating the formation of three circRNAs, Tru_circ00222_001, Tru_circ00222_002 and Tru_circ00222_004. These three circRNAs are generated by alternative splicing from the same host gene: TERG_00222. **b** Four types of AS events identified in the conidial and mycelial stages. A3SS: alternative 3′ splice site; A5SS: alternative 5′ splice site; IR: intron retained; ES: exon skipping

The correspondence of two or more circRNAs to a single host gene may be due to alternative back-splicing events when these circRNAs share identical splicing acceptors or donors [15]. For example, three circRNAs, Tru_circ00222_001, Tru_circ00222_002 and Tru_circ00222_004, were determined to be derived from the same host gene, TERG_00222 (Gene ID: 10377559), in the current study. Analysis of the circRNA gene structures showed that Tru_circ00222_001 and Tru_circ00222_002 have identical circular ends but that Tru_circ00222_002 includes an extra 504 nt sequence. Tru_circ00222_002 and Tru_circ00222_004 also have identical splicing sites, and the Tru_circ00222_002 sequence is 165 nt longer than the Tru_circ00222_004 sequence (Fig. 5a).

AS is a common event that exists in various organisms, greatly expanding the complexity and diversity of the transcriptome [46]. AS events in circRNAs have also been frequently identified in animals and plants, which may facilitate the diversity of circRNAs [47, 48]. To investigate the existence of circRNA AS (circ-AS) in *T. rubrum*, we searched all the sequenced samples using the CIRI-AS algorithm to identify circ-AS events. A total of 4 types of circ-AS events, including exon skipping (ES), intron retention (IR), alternative 5′ splice site (A5SS) splicing and alternative 3′ splice site (A3SS) splicing, were identified in 101 circRNAs (Additional file 8: Table S7). Among these, 91 circ-AS events were specific to conidia, and 7 circ-AS events were specific to mycelia. Three circ-AS events were common to both stages. A3SS splicing was the most common circ-AS type, accounting for 59.57% of all circ-AS events in conidia and 80.0% of events in mycelia. A5SS splicing, which accounted for 45.74% of events in conidia and 20.0% of events in mycelia, was the next most common type. The other two types of circ-AS were rare and were only identified in the conidial stage; IR accounted for 7.45% of events, and ES accounted for 5.32% of events (Fig. 5b). These results showed that circ-AS events were much more common in conidia than in mycelia. To examine whether this difference was due to the greater number of circRNAs in conidia than mycelia, we calculated the AS ratio in each stage. The ratio of AS events was 2.4% in conidia (94 out of 3980) and 1.3% in mycelia (10 out of 778), suggesting a greater AS ratio in conidia than in mycelia. What caused this different AS ratio between the two stages deserves further investigation. Whether the differences between the two stages and among the four AS types are due to bias in circRNA identification could be resolved with advances in methodologies.

### Construction of a competing endogenous RNA (ceRNA) regulatory network

Studies have shown that circRNAs competitively bind miRNAs to prevent the binding of miRNAs to mRNAs; thus, these circRNAs are considered ceRNAs that interact with target miRNAs to control target gene expression [49, 50]. We constructed a ceRNA network based on the DE-circRNAs identified in our study. Among the 940 DE-circRNAs, 661 DE-circRNAs were predicted to target 140 miRNAs (Additional file 9: Table S8), and these miRNAs further regulated 2753 mRNAs (Additional file 10: Table S9). In this network, 207 circRNAs targeted single miRNAs. However, most circRNAs targeted two or more miRNAs, and each miRNA was frequently targeted by more than one circRNA. For example, the circRNA Tru_circ_sc2.10_00071 was predicted to target the most miRNAs (20), followed by Tru_circ_sc2.9_00086 (14 miRNAs) and Tru_circ_sc2.2_00112 (13 miRNAs). In addition, the miRNA Tru-miR-4459 was targeted by the most circRNAs (52), followed by Tru-miR-854 (46 circRNAs) and Tru-miR-762 (45 circRNAs).

Similarly, a single miRNA could target multiple mRNAs. For example, the miRNA Tru-miR-4459 targeted the most mRNAs (406), followed by Tru-miR-595 (354 mRNAs) and Tru-miR-3620-5p (330 mRNAs).

**Fig. 6:**
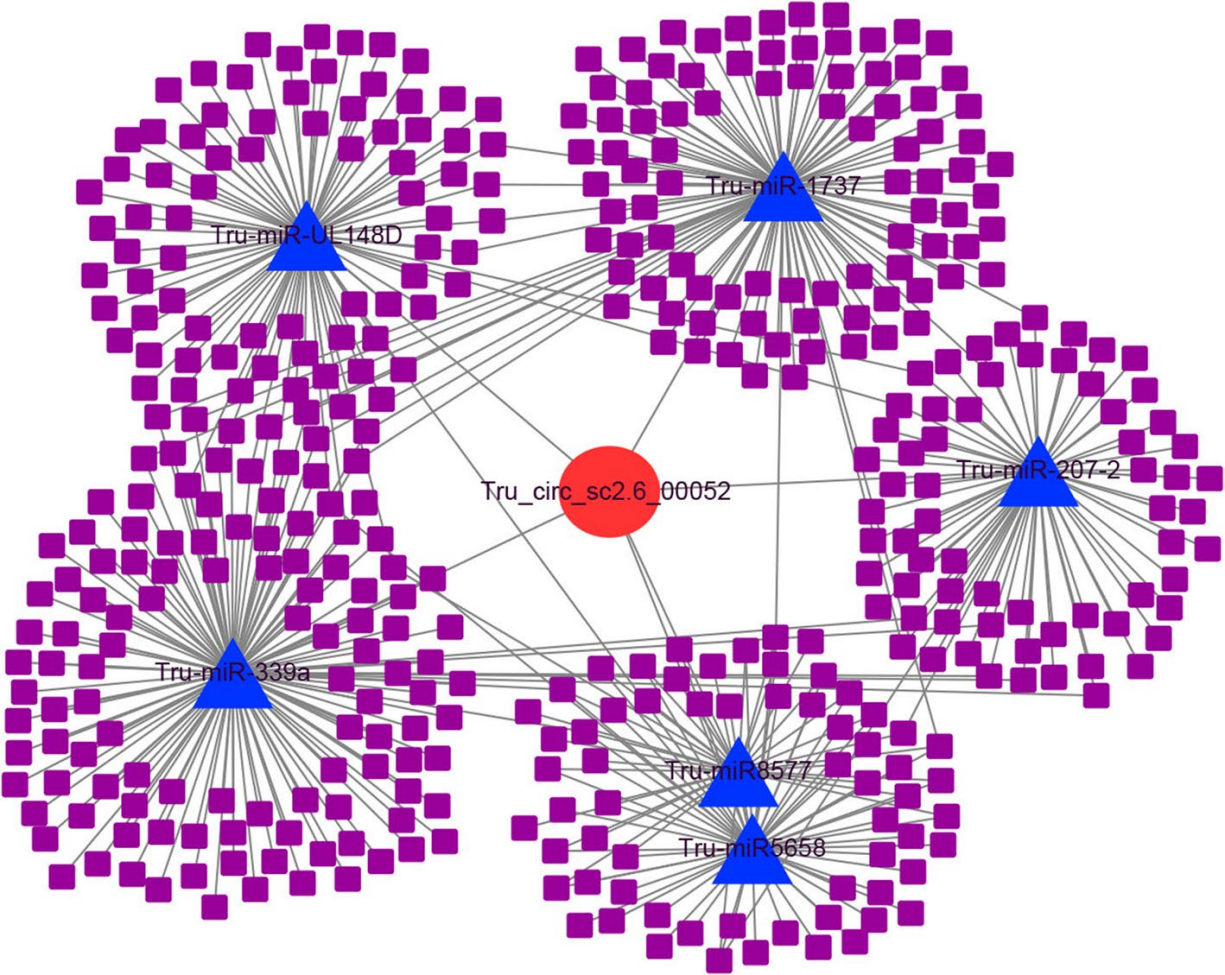
CeRNA network of circRNA Tru_circ_sc2.6_00052. The red circle represents circRNA; the blue triangle represents miRNA; and the purple square represents mRNA

The up-regulated circRNA Tru_circ_sc2.6_00052 was arbitrarily selected to construct a circRNA-miRNA-mRNA network. As shown in Fig. 6, the circRNA Tru_circ_sc2.6_00052 targeted six miRNAs, and these miRNAs further regulated 414 mRNAs in total.

These results illustrate the complexity of gene expression regulation. In addition, transcription factors (TFs) have been found to participate in this regulatory process [51]. In our previous study, the miRNA Tru-miR5658 was suggested to bind to three TFs, including the C6 transcription factor, bZIP transcription factor and transcription factor TFIIIB component [52]. In this study, Tru-miR5658 was targeted by 17 up-regulated circRNAs. This result implies that TFs might associate with circRNAs and miRNAs to regulate target gene expression during *T. rubrum* development.

### Functional annotation of target genes regulated by DE-circRNAs in the ceRNA network

**Fig. 7:**
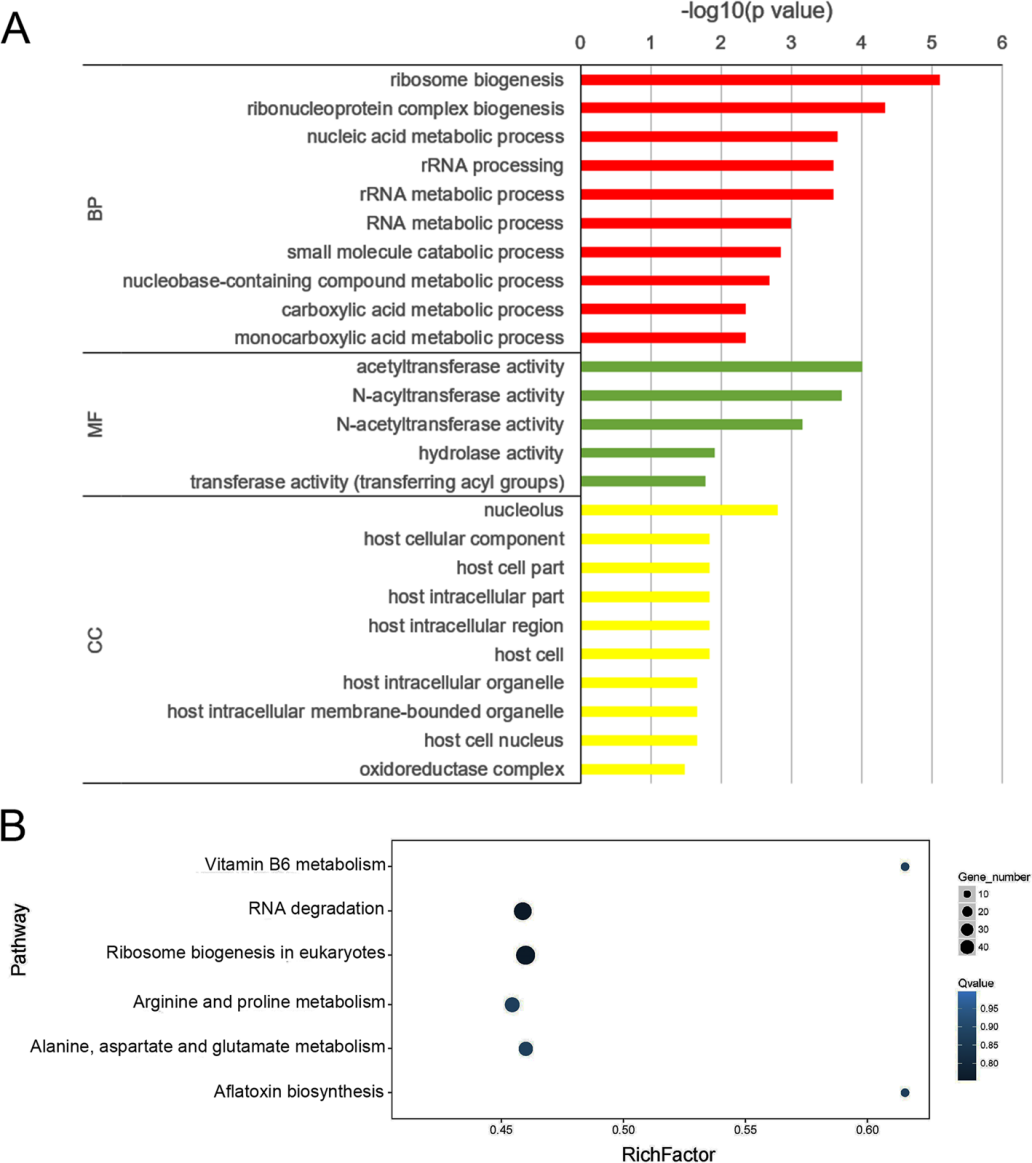
Functional analysis of target genes for DE-circRNAs. **a** GO enrichment of target genes based on biological processes (BP), molecular function (MF) and cellular component (CC). **b** KEGG analysis of target genes

To further uncover the potential roles of circRNAs in the ceRNA network, 2753 target genes regulated by DE-circRNAs were subjected to GO and KEGG enrichment. Based on GO annotation of biological processes, the target genes were heavily involved in ribosome biogenesis, ribonucleoprotein complex biogenesis, rRNA processing, rRNA metabolic processes, and RNA metabolic processes, suggesting their roles in posttranscriptional regulation and protein expression-related pathways. In addition, the target genes participated in small-molecule metabolism, such as small-molecule catabolic processes, nucleobase-containing compound metabolic processes, carboxylic acid metabolic processes and monocarboxylic acid metabolic processes. In the molecular function category, the target genes were significantly involved in acetyltransferase activity and N-acyltransferase activity. In the cellular component category, target genes were significantly associated with the nucleolus, host cellular components and host cell parts (Fig. 7a, Additional file 11: Table S10).

KEGG analysis suggested that the target genes were highly involved in protein synthesis-related processes, including RNA degradation and ribosome biogenesis in eukaryotes. Some small-molecule metabolic processes were also enriched, including arginine and proline metabolism, alanine, aspartate and glutamate metabolism, aflatoxin biosynthesis and vitamin B6 metabolism (Fig. 7b, Additional file 12: Table S11). These results are consistent with the results of GO enrichment analysis in the biological process category stated above.

In addition, some target genes are considered to be related to fungal virulence and pathogenicity, including secreted proteases and multidrug efflux transporters (Additional file 13: Table S12). Since dermatophytes are almost always localized in keratinized tissues, proteolytic enzymes and other elements involved in keratin biodegradation are essential virulence factors for these fungi [53–55]. In our study, 31 target genes were considered secreted proteases or peptidases, such as alkaline phosphatase, aspartyl aminopeptidase, extracellular aspartic endopeptidase, subtilisin-like protease and tripeptidyl peptidase SED3. The multidrug efflux pump, which contributes to antifungal resistance, is considered another factor of fungal virulence [56]. Two main families belong to efflux transporters: the major facilitator superfamily (MFS) and ATP-binding cassette (ABC) superfamily [56, 57]. In total, 3 MFS and 6 ABC superfamily transporters were identified in our study.

### Investigation of the relative expression levels of two circRNA-miRNA-mRNA axes

**Fig. 8:**
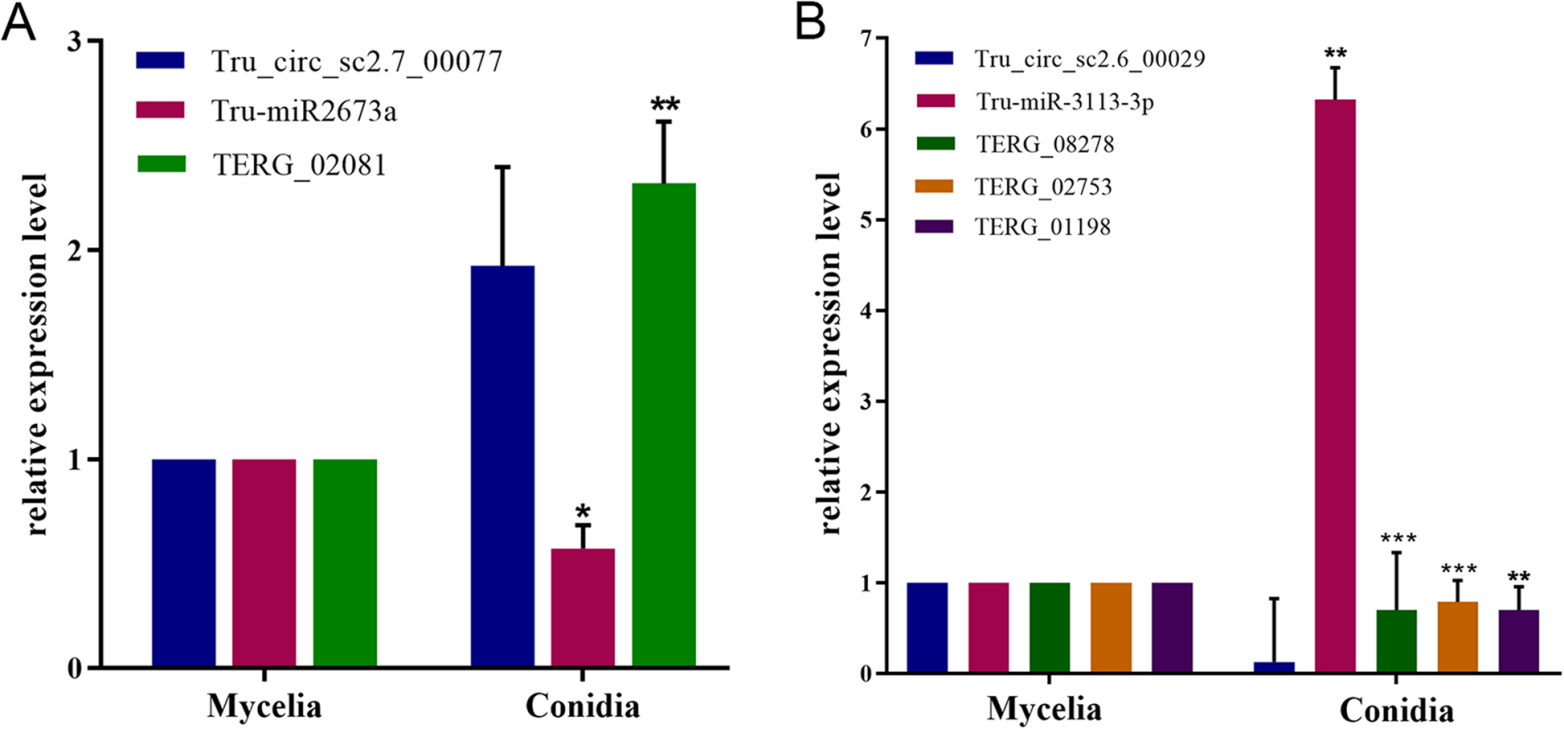
Relative expression levels of two circRNA-miRNA-mRNA axes between the conidial and mycelial stages. **a** The axis of circRNA Tru_circ_sc2.7_00077 - miRNA Tru-miR2673a - target gene TERG_02081. **b** The axis of the circRNA Tru_circ_sc2.6_00029 - miRNA Tru-miR-3113-3p - target genes TERG_08278, TERG_02753 and TERG_01198. *** indicates *p* < 0.001, ** indicates *p* < 0.01 and * indicates *p* < 0.05

Two circRNA-miRNA-mRNA axes in the ceRNA network were randomly selected to investigate their relative expression levels. For one axis, the qRT-PCR results showed that the miRNA Tru-miR2673a was down-regulated, whereas the circRNA Tru_circ_sc2.7_00077 and the target gene TERG_02081 (transcript ID: XM_003237312.1, encoding an RNA-binding protein) were both significantly up-regulated in conidia vs. mycelia (Fig. 8a). For the other axis, the circRNA Tru_circ_sc2.6_00029 was predicted to target the miRNA Tru-miR-3113-3p, and Tru-miR-3113-3p was suggested to target three target genes, TERG_08278 (transcript ID: XM_003231143.1, encoding a serine/threonine protein kinase), TERG_02753 (transcript ID: XM_003235649.1, encoding an E3 ubiquitin-protein ligase) and TERG_01198 (transcript ID: XM_003239168.1, encoding the pre-mRNA splicing factor rse1). Based on the qRT-PCR results, as shown in Fig. 8b, the circRNA Tru_circ_sc2.6_00029 and the three target genes TERG_08278, TERG_02753 and TERG_01198 were all down-regulated in conidia vs. mycelia, while the miRNA Tru-miR-3113-3p was significantly up-regulated. These results together indicate that the circRNA and the target genes are positively associated with each other but that they exhibit an expression trend opposite that of the corresponding miRNA. This finding is consistent with the theory that circRNAs competitively sponge miRNAs to release the corresponding target genes inhibited by miRNAs.

### Conservation of circRNAs in other species

To evaluate circRNA conservation, the circRNAs identified in our study were blasted against circRNAs identified in six animal, plant and nematode model organisms. The results revealed, for example (as shown in Table 3), that 12 circRNAs in *T. rubrum* were homologous to 14 circRNAs in *Mus musculus* and that 9 circRNAs in *T. rubrum* were homologous to 3 circRNAs in *Gallus gallus*. These results suggest that circRNAs are somewhat conserved between *T. rubrum* and these model species.

**Table 3:** Conservation of *T. rubrum* circRNAs in six model organisms

Species	Number of circRNAs	Blasted species	Number of homologous circRNAs
*T. rubrum*	12	*Mus musculus*	14
*T. rubrum*	9	*Gallus gallus*	3
*T. rubrum*	5	*Drosophila melanogaster*	6
*T. rubrum*	7	*Caenorhabditis elegans*	5
*T. rubrum*	14	*Gossypium* spp.	8
*T. rubrum*	7	*Arabidopsis thaliana*	1

**Fig. 9:**
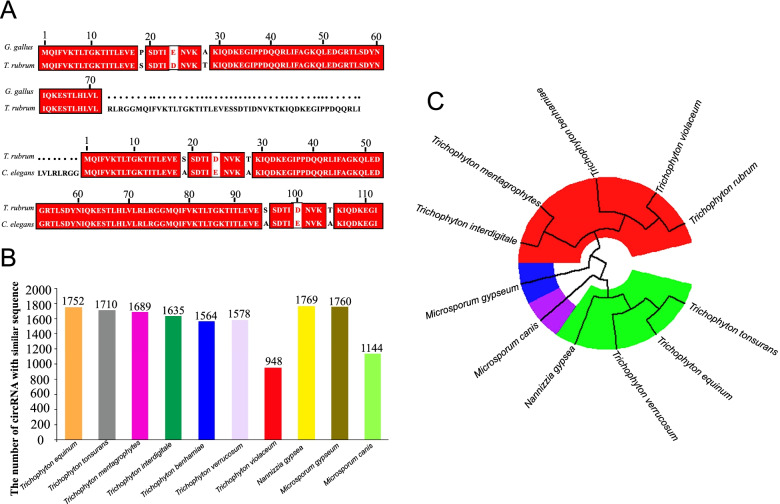
Conservation analysis of *T. rubrum* circRNAs. **a** The protein sequence alignment of host genes between TERG_07138 in *T. rubrum* and UBI in *G. gallus*; TERG_07138 in *T. rubrum* and ubq-1 in *C. elegans.* **b** Conservation of all the identified circRNAs in ten dermatophytes. **c** Phylogenetic tree for Tru_circ07138_001 in *T. rubrum* and ten dermatophytes

Interestingly, the circRNA Tru_circ07138_001 in *T. rubrum* was homologous to circUBI in *G. gallus*, cel_circ_0000387 in *C. elegans* and ghi_circ_000675 in *Gossypium* spp., with nucleotide sequence identity values of 81.08, 94.30 and 81.82%, respectively. The host genes TERG_07138 (Gene ID: 10372121), UBI (Gene ID: 395296) and ubq-1 (Gene ID: 175840), corresponding to three of these circRNAs, Tru_circ07138_001, circUBI and cel_circ_0000387, respectively, all belong to the short-lived polyubiquitin-related gene family, which is involved in the ATP-dependent proteolysis of impaired proteins [22, 58]. In addition, each of these three genes generated only one circRNA. The proteins encoded by these three genes were compared, and the sequence similarity was 95.78% between *T. rubrum* and *G. gallus* and 95.63% between *T. rubrum* and *C. elegans* (E < 1e−5) (Fig. 9a). These results reveal high conservation of the circRNA Tru_circ07138_001 and its host gene in some distantly related species.

We further explored the conservation of circRNAs between *T. rubrum* and other dermatophytes. All circRNAs identified in *T. rubrum* were blasted against the genomes of ten dermatophytes. The results showed that 1752, 1710, 1689, 1635, 1564, 1578, 948, 1769, 1760 and 1144 identified circRNAs in *T. rubrum* were conserved in *Trichophyton equinum, Trichophyton tonsurans, Trichophyton mentagrophytes, Trichophyton interdigitale, Trichophyton benhamiae, Trichophyton verrucosum, Trichophyton violaceum, Nannizzia gypsea, Microsporum gypseum* and *Microsporum canis,* respectively (Fig. 9b). Homologous circRNAs accounted for 22–42% of all circRNAs identified in *T. rubrum*. Furthermore, 162 circRNAs were conserved in all ten dermatophytes, especially the circRNA Tru_circ07138_001 mentioned above. We annotated and visualized a phylogenetic tree for Tru_circ07138_001 in *T. rubrum* and the homologous sequences in these ten dermatophytes (Fig. 9c). Among these dermatophyte species, Tru_circ07138_001 was most conserved in *T. violaceum*, *T. benhamiae*, *T. mentagrophytes* and *T. interdigitale*. However, specificity was also observed. A total of 988 circRNAs were identified only in *T. rubrum,* suggesting that they play specific roles in this particular species.

## Discussion

CircRNAs are a group of ncRNAs, and their essential roles have been gradually revealed. CircRNAs exhibit specific expression patterns in distinct species, tissues, cell types and developmental stages, so they are considered potential biomarkers for the diagnosis of clinical diseases [8, 59–63]. For example, Li et al. identified abundant circRNAs in human serum exosomes, and these serum exosomal circRNAs can be used to distinguish patients with cancer from healthy controls, suggesting that this type of circRNA can be developed into a promising biomarker for cancer diagnosis [64].

In addition, because circRNAs function as important transcription regulators, investigating the roles of circRNAs will facilitate understanding of the biological and physical features of distinct species [65]. Although the essential roles of circRNAs have been suggested in many organisms, the characteristics of fungal circRNAs are largely unknown. *T. rubrum* is considered a model organism of dermatophytes and human pathogenic filamentous fungi. In our study, circRNAs in *T. rubrum* were first identified with whole-genome RNA-seq. A total of 4254 circRNAs were identified, and most of them were developmental stage-specific. This pattern is consistent with the results observed in most other species [28, 66]. Among these, 940 circRNAs were significantly differentially expressed between the two stages. PCR confirmed the circularized structures and relative expression levels of randomly selected circRNAs, supporting the reliability of our data.

Based on the genomic distribution of circRNAs, 60.5% were derived from intergenic regions, while the others were derived from exonic (35.2%) and intronic regions (4.3%). This distribution pattern in *T. rubrum* is different from those in some animals and plants in which most circRNAs are derived from intronic and exonic regions [67–69]. In contrast, in some fungal species, such as *M. oryzae* and *A. apis*, circRNAs are derived mainly from intergenic regions, displaying distribution patterns similar to those of *T. rubrum* circRNAs [27, 28]. The differences in circRNA origin among fungi, animals and plants may be due to the distinct characteristics of circRNAs in different kinds of organisms [70]. In addition, the quality of genome annotation might be an important factor influencing the results of circRNA location. For instance, erroneously divided genes and missing genes in the annotation would increase the proportion of circRNAs in the intergenic regions. Whether the difference in circRNA distribution between *T. rubrum* and animals and plants is affected by the current computational approach-based annotation would be elucidated with further improved annotation of the *T. rubrum* genome.

Among all the identified circRNAs, 1680 circRNAs mapped to 1055 host genes. In some cases, two or more circRNAs stemmed from one host gene. Some of these instances may have been due to AS events. Four types of circ-AS events corresponding to 101 circRNAs were identified in our study, including ES, IR, A5SS splicing and A3SS splicing events. Among these, A3SS is the most prevalent splicing event, and ES is the rarest. This AS pattern of circRNAs is different from that of linear mRNAs, of which IR is the most common AS type in fungi [71–74]. AS of both circRNA and mRNA is regulated by RNA-binding proteins and splicing factors [1, 75–77]. However, it has been proven that these regulating factors in circRNA binding sites are different from those in linear mRNA binding sites [15]. The distinct preference of regulating factors for circRNA and mRNA might be a possible reason for the difference in their splicing patterns. Furthermore, we found that DE-circRNAs derived from identical host genes showed consistent relative expression levels in conidia vs. mycelia. Whether circRNAs derived from common host genes have the same expression trends and whether interactions occur between circRNAs and their host genes need to be investigated further. Some studies have suggested that circRNAs compete with their host pre-mRNAs, but others have suggested that a subclass of circRNAs (exon-intron circRNAs) enhance the transcription of their host genes [78, 79]. The specific relationships between circRNAs and their host genes should be explored further.

It is widely accepted that circRNAs serve a regulatory role by acting as miRNA sponges and further facilitating mRNA expression, thus constructing a complex ceRNA network [80, 81]. We depicted a regulatory network of DE-circRNAs in which 661 DE-circRNAs targeted 140 miRNAs and further regulated 2753 mRNAs. In this network, one circRNA could target multiple miRNAs, and one miRNA might regulate various mRNAs. We randomly selected two circRNA-miRNA-mRNA axes to investigate their expression patterns. The results all showed similar expression trends of circRNAs and mRNAs that were opposite those of miRNAs, supporting the ceRNA theory. Similar results have also been illustrated in other species. For instance, cells of the human monocyte line THP-1 infected by *Trichosporon asahii* (*T. asahii*) were compared with uninfected samples, and a differentially expressed ceRNA network was constructed [82]. The relative expression level of a circRNA-miRNA-mRNA axis was investigated by RT-qPCR. The results showed that circRNA hsa_circ_0065336 and target gene PTPN11 were both up-regulated after infection with *T. asahii*, whereas miRNA miR-505-3p was down-regulated [82]. In another instance, circRNA hsa_circ_0007874 was down-regulated in hepatocellular carcinoma (HCC) compared with normal controls, and its related ceRNA pathway hsa_circ_0007874-miR-9-CDX2 was tested with luciferase assays [83]. The results showed that the luciferase activity with the target gene CDX2 decreased dramatically with the increase of miRNA miR-9. However, after circRNA hsa_circ_0007874 was overexpressed, the decline in luciferase activity was repressed [83]. All of these results are consistent with the ceRNA theory, suggesting that circRANs could indirectly regulate target gene expression by sponging miRNAs [82].

Other regulatory factors, such as TFs, may also be involved in the regulation of gene expression based on a ceRNA regulating mechanism [84]. A miRNA could target a TF, and a TF binds to the specific sequence of the enhancer or promoter region of the DNA, thereby suppressing or activating target gene expression in the transcriptional process [84, 85]. For instance, in spinal cord injury, miRNA miR-674-5p targets a TF Stat1, which regulates the expression of many target genes [86]. Similarly, in our study, miRNA in the ceRNA network was also suggested to target TFs. These processes indicate the complexity of the network that regulates gene expression.

Functional annotation of the target genes suggested that some of them are involved in various small-molecule metabolic processes. Moreover, the target genes were found to be heavily involved in biogenesis-, processing- and metabolism-related processes of ribosomes and RNA, suggesting that these target genes might participate in posttranscriptional processes and protein synthesis. Based on these findings, we speculate that circRNAs might interact with these target genes to affect gene expression at the posttranscriptional and translational levels in addition to acting as miRNA sponges to release mRNA, thus facilitating protein expression. Furthermore, some target genes were identified as secreted proteases and multidrug efflux transporters, suggesting that circRNAs might play a role in the virulence of this fungus.

CircRNAs are highly conserved in mammals [87]. Approximately 25–40% of circRNAs in humans are homologous to corresponding genomic regions in mice [88]. In addition, 29% of zebrafish circRNAs show homology with human and mouse circRNAs [21]. In plants, 40 and 8% of circRNAs identified in *A. thaliana* are orthologous to circRNAs in *Oryza sativa* and *Solanum lycopersicum,* respectively [89]. In our study, we found that circRNAs in *T. rubrum* exhibited rare conservation with circRNAs in some animal, plant and nematode model organisms that fewer than 1% of the circRNAs were conserved in these species. This low conservation may be due to the distant phylogenetic relationships between *T. rubrum* and these other species. In contrast, circRNAs in *T. rubrum* were relatively highly homologous to the corresponding genomic region in closely related dermatophytes, accounting for 22–42% of all identified circRNAs. In particular, the circRNA Tru_circ07138_001 was conserved in all dermatophyte species analyzed in our study. In addition, this circRNA was conserved in three distantly related species *G. gallus*, *C. elegans* and *Gossypium* spp.*,* and its host gene TERG_07138 was highly homologous to the corresponding circRNA host genes in *G. gallus* and *C. elegans*. Interestingly, these three homologous host genes all belong to the polyubiquitin-related family. All of these results demonstrate high conservation of this circRNA and its host gene in these different species, suggesting that further exploration of the potential function and role of this conserved circRNA is warranted.

## Conclusions

Our research provides the first global profile of circRNAs in *T. rubrum* and dermatophytes. These data could lay a foundation for further study on circRNA regulatory mechanisms and provide valuable clues for in-depth exploration of the biological characteristics of *T. rubrum* and closely related species. Such pursuits will facilitate the fight against these significant human fungal pathogens.

## Methods

### Strain culture and sample collection

*T. rubrum* strain BMU 01672 was incubated on potato dextrose agar medium (Becton Dickinson, Sparks, MD, USA) at 28 °C for 15 days to induce conidia formation. Conidia were collected with distilled water at 4 °C and then filtered twice through Miracloth (Merck, Billerica, MA, USA) to remove mycelial fragments. Conidial purity was examined with a microscope. To obtain mycelia, 1 × 10^6^ conidia were inoculated into 100 ml YPD liquid medium (Becton Dickinson) and cultured at 28 °C under constant shaking (180 rpm) for 72 h [90]. The medium was removed by thorough washing with cold distilled water, and mycelia were collected through centrifugation at 4000×*g* for 5 min.

### RNA extraction and library sequencing

Three biological replicates were performed for each conidial and mycelial stage, and 300 mg material was used for each replicate. Total RNA was extracted using TRIzol reagent (Invitrogen, Carlsbad, CA, USA) according to the manufacturer’s protocol. Then, RNase-free DNase (Qiagen, Hilden, Germany) was added to eliminate potential genomic DNA contamination. The integrity and purity of total RNA were assessed with an Agilent 2100 Bioanalyzer system (Agilent Technologies, Santa Clara, CA, USA) and a NanoDrop ND-1000 spectrophotometer (Thermo Scientific, Waltham, MA, USA).

In each sample, rRNA was removed using a Ribo-Zero rRNA Removal Kit (Epicentre, Madison, WI, USA), and linear RNA was digested with RNase R (Epicentre). The remaining RNA was fragmented, and the fragments were reverse-transcribed to construct cDNA libraries following the protocol of an NEBNext Ultra Directional RNA Library Prep Kit for Illumina (NEB, Ipswich, MA, USA). After repairing and filling the ends of double-stranded cDNA, NEBNext adaptors were ligated. cDNA fragments of 150–200 bp were selected and purified using AMPure XP Beads (Beckman Coulter, Beverly, MA, USA). Afterward, the ligated products were PCR-amplified, and the library quality and quantity were evaluated with an Agilent Bioanalyzer 2100 system and qRT-PCR. Finally, 150 bp paired-end reads were sequenced on the Illumina HiSeq™ 4000 platform.

### CircRNA identification and differential expression analysis

Adaptors and low-quality reads that contained poly-N sequences or had low Q scores were removed to obtain clean reads, and the Q20, Q30, GC content and sequence duplication level were calculated. The clean reads were mapped to the *T. rubrum* reference genome using BWA software to identify junction reads [91]. These junction reads were realigned to the *T. rubrum* reference genome to determine the splice sites. The junction reads with paired chiastic clipping (PCC) signals and paired-end mapping (PEM) sites were considered high-confidence back-spliced junction reads. In addition, only the back-spliced junction reads flanking the GU/AG splice signal were considered further. CircRNAs were identified using the CIRI tool based on a cutoff value of back-spliced junction reads ≥2 [9]. We also used CircRNA Identifier-Alternative Splicing (CIRI-AS) software to identify AS events in circRNAs [15].

The spliced reads per billion mapping (SRPBM) values were calculated to normalize the circRNA reads in each sample, and the number of junction reads per circRNA was determined with Circos software. The differential expression values of circRNAs were calculated using NOIseq, and DE-circRNAs were identified with the thresholds of |log_2_ (fold change)| ≥1 and probability ≥0.8 [92, 93].

### Gene function annotation and ceRNA network construction

Gene function annotation was performed based on GO terms using the clusterProfiler R package [94] and KEGG (https://www.kegg.jp/) [95–97]. MiRNA target sites of circRNAs were predicted with the miRanda program based on the conserved seed-matching sequence [98], and mRNAs targeted by miRNAs were identified using TargetFinder software [99]. Based on the theory of ceRNA, we constructed a circRNA-miRNA-mRNA interaction network using Cytoscape 3.5.1 software [100].

### Conservation analysis of *T. rubrum* circRNAs

Conservation of circRNAs was investigated in six distantly related species and ten closely related dermatophytes. CircRNAs identified in *T. rubrum* were blasted against circRNAs of six distantly related species, including *G. gallus*, *D. melanogaster*, *Gossypium* spp., *A. thaliana*, *C. elegans,* and *M. musculus,* using BlastN with the criteria of score > 50, identity> 50%, and E value <1e^− 5^. The circRNAs of these six species were deposited in the circBase database (http://www.circbase.org/) and the CircFunBase database (http://bis.zju.edu.cn/CircFunBaseBlast/). Genome sequences of ten dermatophytes, including *T. equinum* CBS 127.97, *T. tonsurans* CBS 112818, *T. mentagrophytes* D15P127, *T. interdigitale* MR816, *T. benhamiae* CBS 112371, *T. verrucosum* HKI 0517, *T. violaceum* CMCC (F) T31, *N. gypsea* CBS 118893, *M. gypseum* CBS 118893 and *M. canis* CBS 113480*,* were downloaded from NCBI (https://www.ncbi.nlm.nih.gov/). The genome sequences of all circRNAs identified in *T. rubrum* were then compared with these dermatophyte genomes using BlastN (v2.11.0, E < 1e^− 5^). A phylogenetic tree of circRNAs was visualized and annotated using the ggtree R package [101]. Multiple sequence alignments were conducted using the ClustalW web server with the default settings [102].

### Validation of circRNA circularization by PCR and Sanger sequencing

Total RNA was treated with DNase I and RNase R, and the remaining RNA was reverse-transcribed into cDNA with random primers using a SuperScript® III First-Strand Synthesis System (Invitrogen) following the manufacturer’s protocol. Genomic DNA was extracted using a DNeasy Plant Mini Kit (Qiagen). Divergent primers and convergent primers of seven randomly selected DE-circRNAs were designed using Primer-BLAST (http://www.ncbi.nlm.nih.gov/tools/primer-blast) according to the methods in previous studies [68, 87]. All primer sequences are provided in Additional file 14: Table S13. The PCR procedure was as follows: 94 °C for 1 min; 35 cycles of 98 °C for 10 s, annealing at 52 °C for 30 s and extension at 72 °C for 12 s; and a final extension at 72 °C for 10 min. The PCR products were detected using 2% agarose gel electrophoresis. The expected bands were excised, and the DNA was eluted using a QIAquick gel extraction kit (Qiagen). Sanger sequencing was performed to further verify the existence of the back-spliced junction sites of these circRNAs.

### Validation of circRNA relative expression levels

The relative expression levels of the seven DE-circRNAs illustrated above were validated by qRT-PCR with divergent primers (Additional file 14: Table S13). qRT-PCR was performed on an ABI 7500 Real-Time PCR Detection System (Applied Biosystems, Waltham, MA, USA) using PowerUp™ SYBR™ Green Master Mix (Applied Biosystems). The conditions were as follows: initial denaturation for 2 min at 95 °C followed by 40 cycles of 15 s at 95 °C, 15 s at 58 °C, and 60 s extension at 72 °C. The relative expression level was calculated using the 2^−ΔΔCt^ method [103]. Data were normalized with two reference genes, DNA-dependent RNA polymerase II (rpb2) and chitin synthase (chs1), as previously described [104]. Statistical significance was determined with GraphPad Prism 8 software (GraphPad Software Inc., San Diego, CA, USA). *p* < 0.05 was considered to indicate significance.

### Detection of the relative expression levels of two circRNA-miRNA-mRNA axes

We randomly selected two circRNA-miRNA-mRNA axes and examined the relative expression levels of the circRNAs, miRNAs and mRNAs in these axes with respect to the conidial and mycelial stages. The primer sequences are listed in Additional file 14: Table S13. qRT-PCR of mRNA was performed with reagents and procedures identical to those described above for circRNAs. The procedure for miRNA extraction was as follows: total RNA was extracted using a miRNeasy Mini Kit (Qiagen), and gDNA was eliminated using a TURBO DNA-free™ Kit (Thermo Fisher Scientific) at 37 °C for 30 min. MiRNA was then reverse-transcribed with a miRcute Plus miRNA First-Strand cDNA kit (TIANGEN, Beijing, China) in a 20 μl reaction volume at 42 °C for 60 min and 95 °C for 3 min. qRT-PCR of miRNA was performed using miRcute SYBR Green Master Mix (TIANGEN). The reaction conditions were 15 min at 95 °C followed by 40 cycles of 20 s at 94 °C and 34 s at 60 °C. U6 was used as the internal control of miRNAs for gene expression normalization. Each experiment was performed with three replicates. The relative expression level and statistical significance were calculated as stated above.

## Supplementary Information

**Additional file 1: Figure S1-S8**. All supplementary figures.

**Additional file 2: Table S1**. CircRNAs identified in conidial and mycelial stage of *T. rubrum*.

**Additional file 3: Table S2**. Significant differentially expressed circRNAs in conidial vs. mycelial stage.

**Additional file 4: Table S3**. GO classification of host genes for all the identified circRNAs.

**Additional file 5: Table S4**. KEGG pathway analysis of host genes for all the identified circRNAs.

**Additional file 6: Table S5**. GO classification of host genes for DE-circRNAs.

**Additional file 7: Table S6**. KEGG pathways analysis of host genes for DE-circRNAs.

**Additional file 8: Table S7**. CircRNA AS events identified in *T. rubrum* conidial and mycelial stages.

**Additional file 9: Table S8**. MiRNAs targeted by DE-circRNAs.

**Additional file 10: Table S9**. mRNAs targeted by miRNAs.

**Additional file 11: Table S10**. GO enrichment of target genes for DE-circRNAs.

**Additional file 12: Table S11**. KEGG pathways analysis of target genes for DE-circRNAs.

**Additional file 13: Table S12**. The target genes related to fungal pathogenicity.

**Additional file 14: Table S13**. Primers used in this study.

## Data Availability

The data supporting the conclusions of this article are within the paper and its additional files. The sequence data generated from *T. rubrum* conidia and mycelia has been deposited in the NCBI SRA database with the accession number PRJNA701864, which can be accessed with https://www.ncbi.nlm.nih.gov//bioproject/PRJNA701864.

## Abbreviations

A3SS: Alternative 3′ splicing site
A5SS: Alternative 5′ splicing site
AS: Alternative splicing
CircRNA: Circular RNA
circ-AS: Circular RNA alternative splicing
ceRNA: Competing endogenous RNA
DE-circRNA: Differentially expressed circRNA
IR: Intron retention
miRNA: microRNA
ncRNA: Non-coding RNA
pre-mRNA: Precursor mRNA
RNA-seq: High-throughput RNA sequencing
SE: Skipping exon
SRPBM: Spliced reads per billion mapping
TFs: Transcription factors
*T. rubrum*: *Trichophyton rubrum*
