# Supplementary material for: Genome-wide identification and functional analysis of circRNAs in Trichophyton rubrum conidial and mycelial stages
Source: BMC Genomics. 2022 Jan 4;23:21. doi: 10.1186/s12864-021-08184-y (PMC8725419; doi:10.1186/s12864-021-08184-y)
Supplement: Supplementary file 1 — Additional file 1: Figure S1-S8. All supplementary figures. [file 12864_2021_8184_MOESM1_ESM.pdf]

Supplementary Figures and Legends

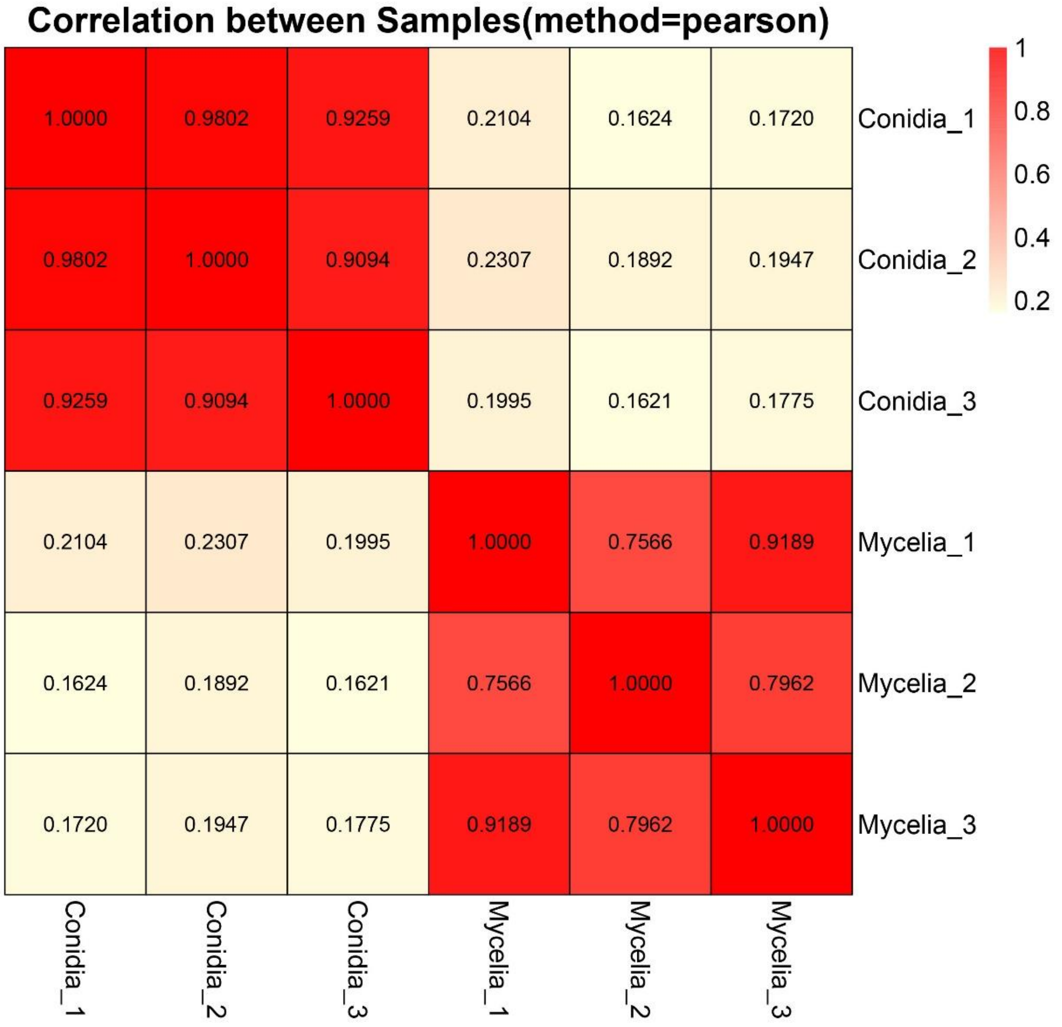

Figure S1. Pearson's correlation coefficients between the conidial and mycelial samples. Three replicates were performed for each the conidial and mycelial stages. X and Y axes represent the samples and each value represents Pearson's correlation coefficient for circRNA expression level.

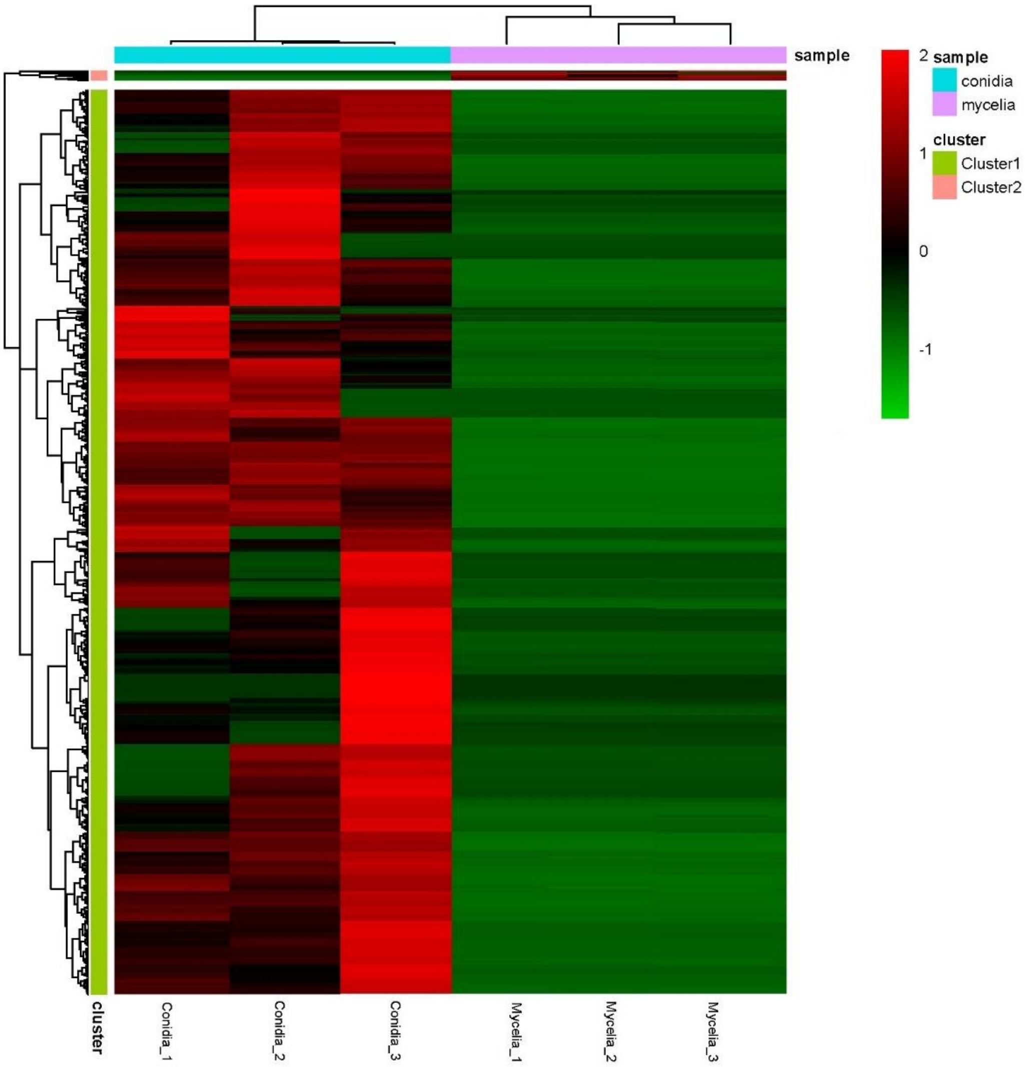

Figure S2. Heat map of expression level for DE-circRNAs in the conidial and mycelial samples. Cluster 1 indicates up-regulated circRNAs, and cluster 2 indicates down-regulated circRNAs in the conidial *vs*. mycelia stage.

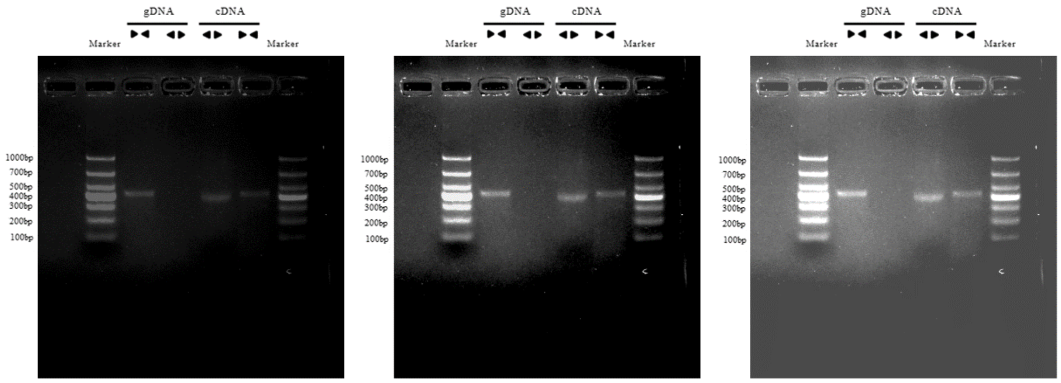

Tru_circ08130_001

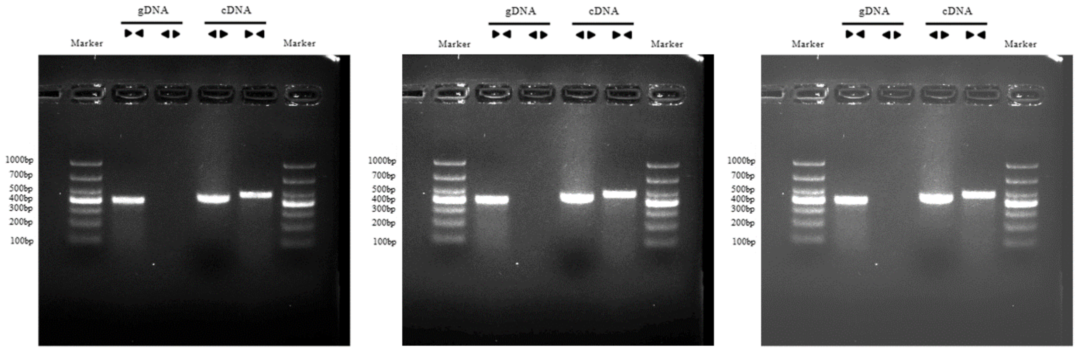

Tru_circ05540_001

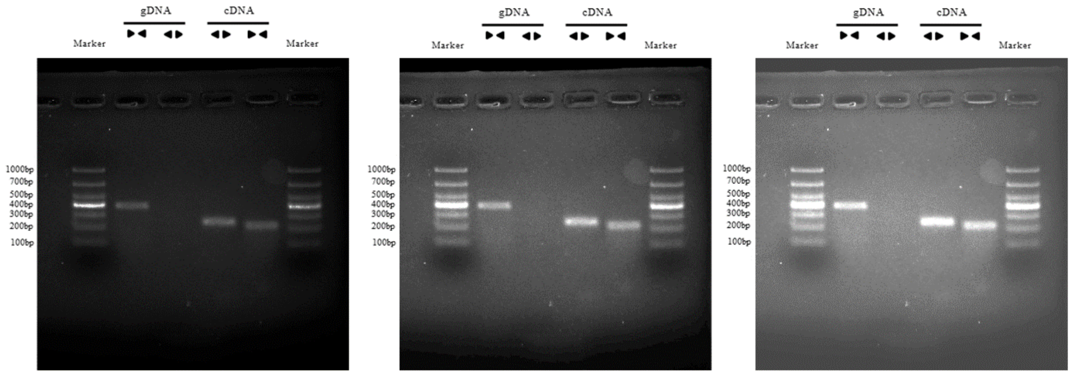

Tru_circ00222_004

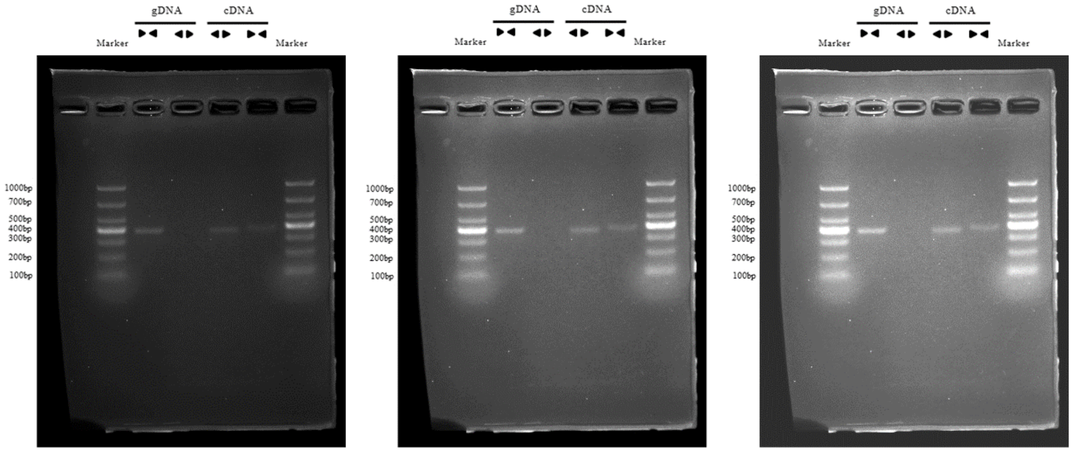

Tru_circ_sc2.6_00052

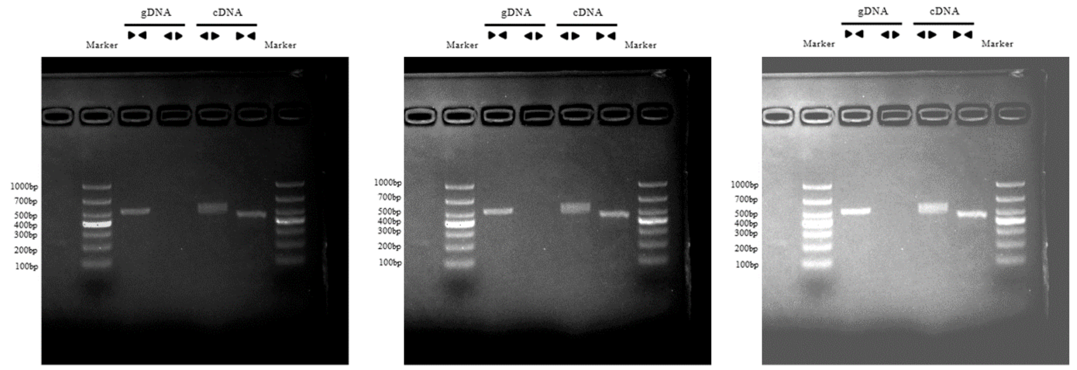

Tru_circ_sc2.5_00073

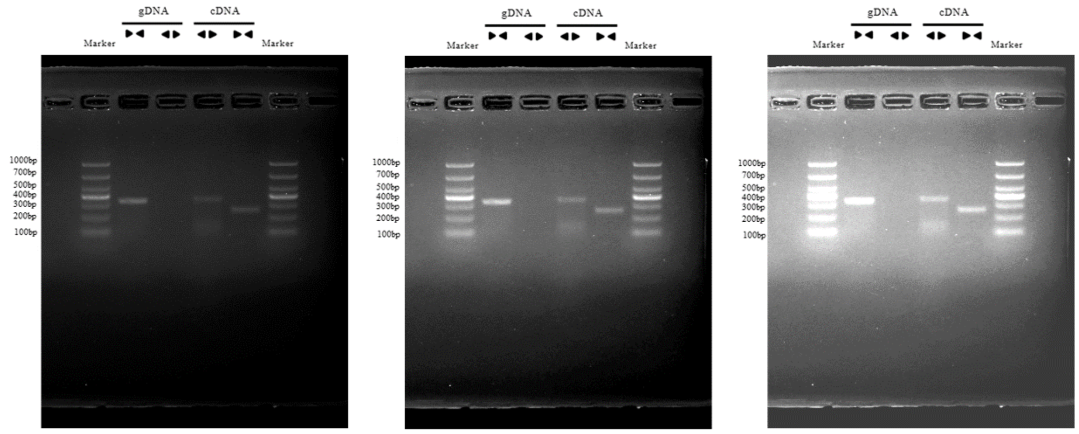

Tru_circ_sc2.2_00045

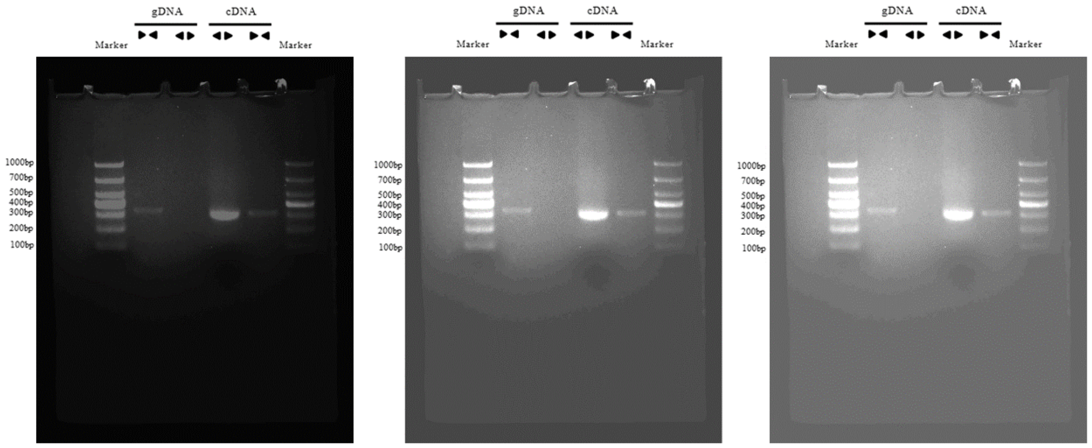

Tru_circ_sc2.1_00115

Figure S3. The uncropped gel with three different contrast corresponding to Fig. 4. Each gel shows the bands that were amplified with divergent and convergent primers of both circRNA and gDAN control.

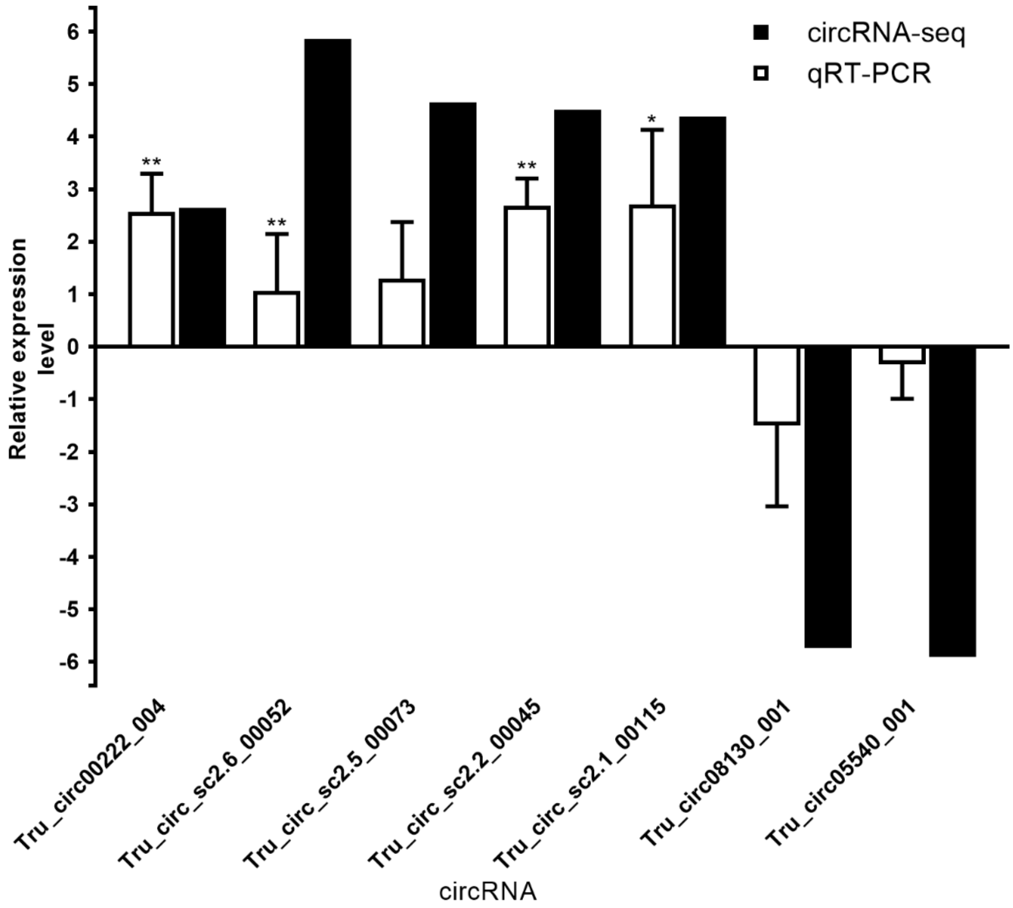

Figure S4. Relative expression level of seven DE-circRNAs based on RNA-seq and qRT-PCR. ** indicates p< 0.01 and * indicates p< 0.05.

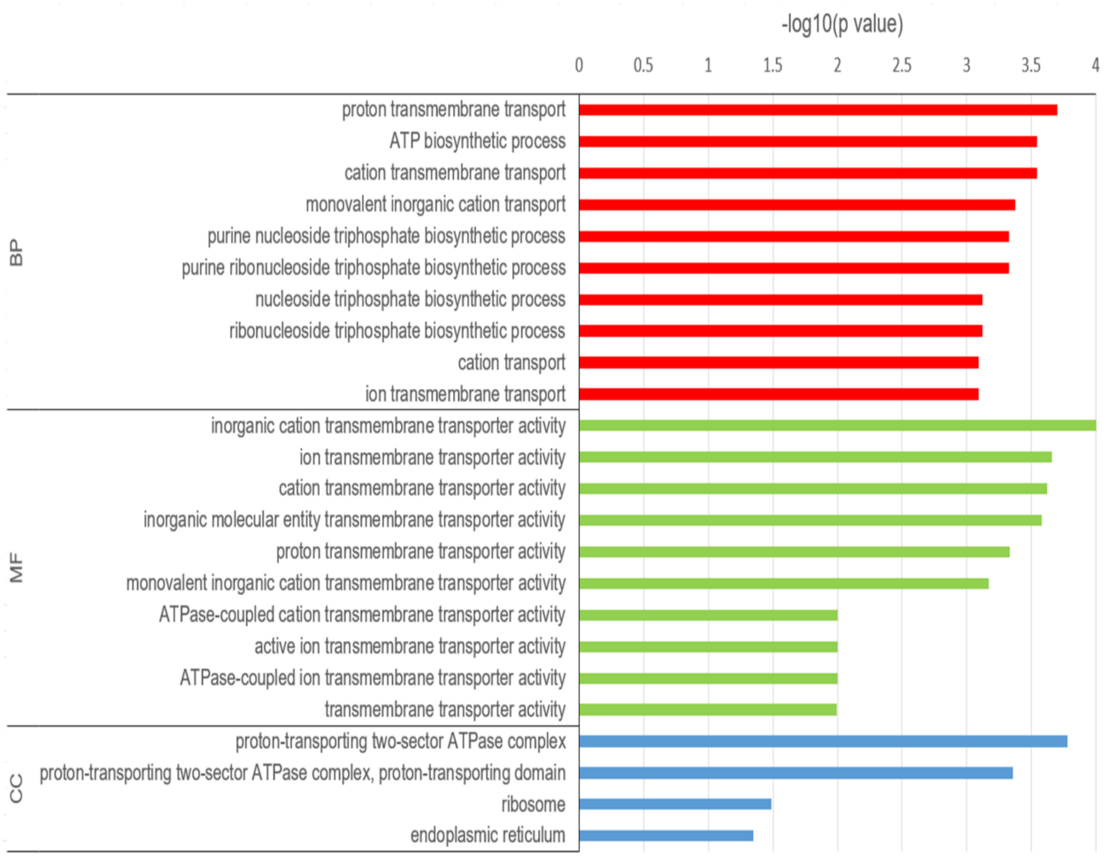

Figure S5. GO enrichment of host genes for all the identified circRNAs in *T. rubrum* (p-value <0.05).

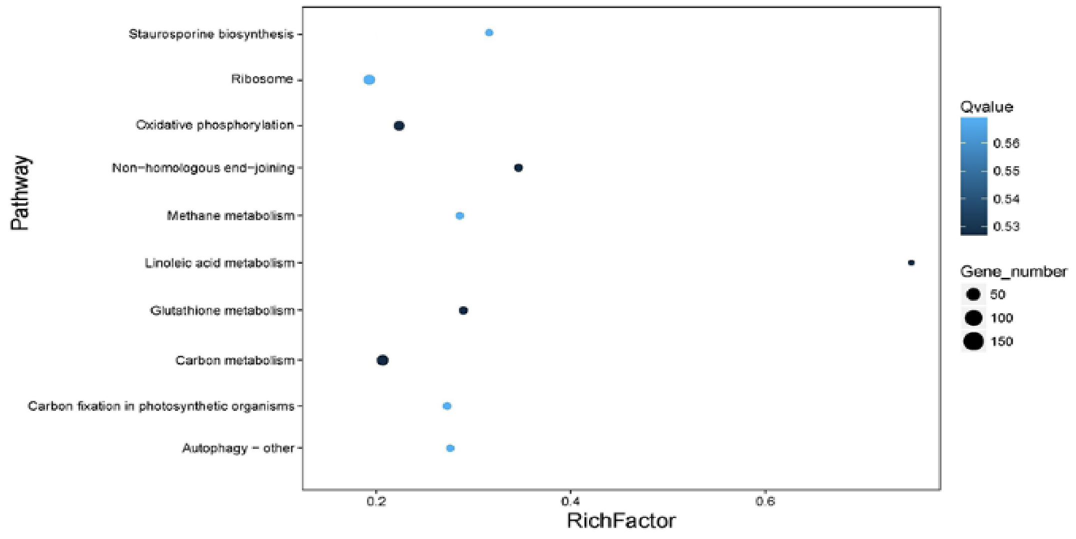

Figure S6. KEGG analysis of host genes for all the identified circRNAs in *T. rubrum* (p-value <0.05).

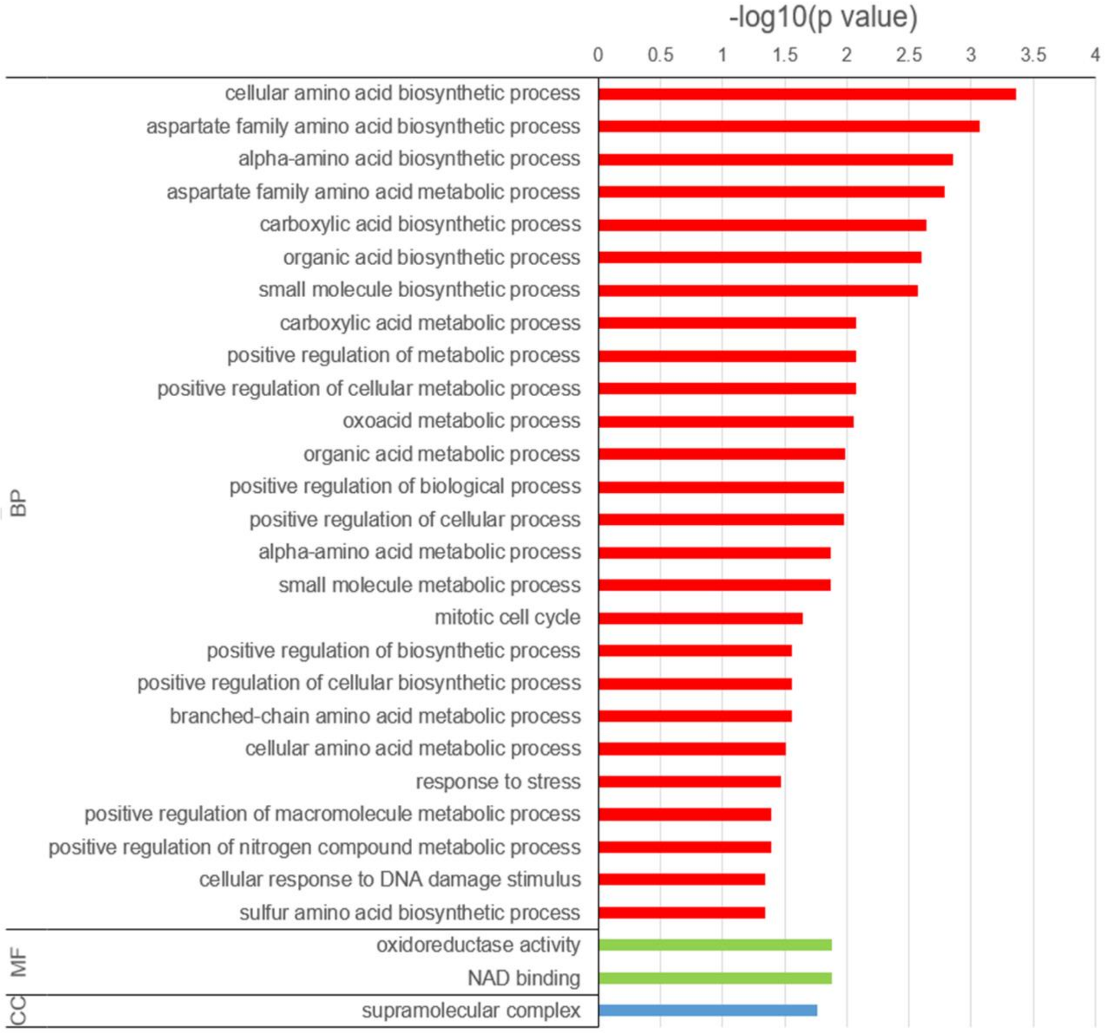

Figure S7. GO enrichment of host genes for DE-circRNAs in *T. rubrum* (p-value <0.05).

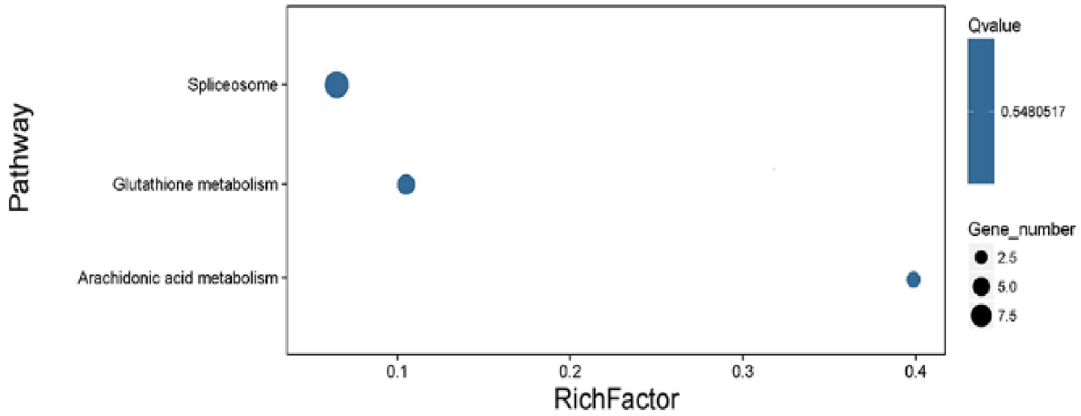

Figure S8. KEGG analysis of host genes for DE-circRNAs in *T. rubrum* (p-value <0.05).
